# Exploring the Efficiency of Algerian Kaolinite Clay in the Adsorption of Cr(III) from Aqueous Solutions: Experimental and Computational Insights

**DOI:** 10.3390/molecules29092135

**Published:** 2024-05-04

**Authors:** Karima Rouibah, Hana Ferkous, Meniai Abdessalam-Hassan, Bencheikh Lehocine Mossab, Abir Boublia, Christel Pierlot, Amdjed Abdennouri, Ivalina Avramova, Manawwer Alam, Yacine Benguerba, Alessandro Erto

**Affiliations:** 1 Laboratory of Materials-Elaborations-Properties-Applications, Department of Process Engineering, University Mohammed Seddik Benyahia, Jijel 18000, Algeria; karima.rouibah@univ-jijel.dz; 2 Laboratoire de Génie Mécanique et Matériaux, Département de Technologie, Faculté de Technologie, Université 20 Août 1955 de Skikda, Skikda 21000, Algeria; h.ferkous@univ-skikda.dz; 3 Laboratory of Environmental Process Engineering, Faculty of Process Engineering, University of Constantine 3, Constantine 25000, Algeria; abdeslam.meniai@univ-constantine3.dz (M.A.-H.); mossaab.bencheikh@gmail.com (B.L.M.); 4 Laboratoire de Physico-Chimie des Hauts Polymères (LPCHP), Département de Génie des Procèdes, Faculté de Technologie, Ferhat Abbas Setif 1 University, Setif 19000, Algeria; abir.boublia@univ-setif.dz; 5 Laboratoire UCCS-Unité de Catalyse et Chimie du Solide, National Graduate School of Engineering Chemistry of Lille (ENSCL), 59650 Villeneuve-d’Ascq, France; christel.pierlot@univ-lille.fr; 6 Laboratoire de Catalyse, Bioprocédés et Environnement, Université 20 Août 1955 de Skikda, BP 26, Route El Hadaik, Skikda 21000, Algeria; a.abdennouri@univ-skikda.dz; 7 Institute of General and Inorganic Chemistry, Bulgarian Academy of Sciences, Block 11, Acad. G. Bonchev Str., 1113 Sofia, Bulgaria; ivalina@svr.igic.bas.bg; 8 Department of Chemistry, College of Science, King Saud University, P.O. Box 2455, Riyadh 11451, Saudi Arabia; maalam@ksu.edu.sa; 9 Laboratory of Biopharmaceutical and Pharmacotechnical (LBPT), Ferhat Abbas Setif 1 University, Setif 19000, Algeria; yacinebenguerba@univ-setif.dz; 10 Dipartimento di Ingegneria Chimica, Dei Materiali e della Produzione Industriale, Università Di Napoli Federico II, P.le Tecchio 80, 80125 Napoli, Italy

**Keywords:** chromium, adsorption, kaolinite, DFT, COSMO-RS, quantum theory of atoms in molecules (QTAIM)

## Abstract

The current study comprehensively investigates the adsorption behavior of chromium (Cr(III)) in wastewater using Algerian kaolinite clay. The structural and textural properties of the kaolinite clay are extensively characterized through a range of analytical methods, including XRD, FTIR, SEM-EDS, XPS, laser granulometry, N_2_ adsorption isotherm, and TGA–DTA. The point of zero charge and zeta potential are also assessed. Chromium adsorption reached equilibrium within five minutes, achieving a maximum removal rate of 99% at pH 5. Adsorption equilibrium is modeled using the Langmuir, Freundlich, Temkin, Elovich, and Dubinin–Radushkevitch equations, with the Langmuir isotherm accurately describing the adsorption process and yielding a maximum adsorption capacity of 8.422 mg/g for Cr(III). Thermodynamic parameters suggest the spontaneous and endothermic nature of Cr(III) sorption, with an activation energy of 26.665 kJ/mol, indicating the importance of diffusion in the sorption process. Furthermore, advanced DFT computations, including COSMO-RS, molecular orbitals, IGM, RDG, and QTAIM analyses, are conducted to elucidate the nature of adsorption, revealing strong binding interactions between Cr(III) ions and the kaolinite surface. The integration of theoretical and experimental data not only enhances the understanding of Cr(III) removal using kaolinite but also demonstrates the effectiveness of this clay adsorbent for wastewater treatment. Furthermore, this study highlights the synergistic application of empirical research and computational modeling in elucidating complex adsorption processes.

## 1. Introduction

Water pollution remains a critical global challenge impacting both the economic and social sectors, notably affecting public health and agricultural quality. Environmental concern arises from the contamination of water bodies by a large variety of pollutants, including natural and anthropogenic heavy metals, toxic compounds, and persistent organic pollutants. The widespread nature of these contaminants poses significant risks, necessitating urgent attention and effective remediation strategies to safeguard water quality and public health [1,2]. As the industrial and agricultural sectors evolve, they release various pollutants into the environment, including organic and inorganic substances, heavy metals, and other harmful compounds. Heavy metals, known for their persistence and non-biodegradability, can easily enter and accumulate in the food chain, posing significant threats to natural ecosystems and human health. This contamination underscores the need for effective environmental management and pollution control strategies [3,4,5].

Chromium (Cr) is a heavy metal of significant concern commonly found in water and wastewater, primarily existing in two oxidation states: trivalent chromium (Cr(III)) and hexavalent chromium (Cr(VI)) [6,7]. Although Cr(VI) is well-known for its acute toxicity, high mobility, and carcinogenicity, posing substantial threats to public health and ecosystems, the impact of Cr(III) is equally pressing but less warned. Cr(III) is essential for human health in trace amounts but becomes harmful at higher concentrations, where it can disrupt metabolic processes and cause renal, dermatological, and ocular damages. Moreover, Cr(III) has been shown to adversely affect a wide range of organisms, including plants, fish, and crustaceans, at elevated levels, indicating its potential for ecological harm. This evidence underscores the importance of focusing on Cr(III) alongside Cr(VI) for a holistic understanding of chromium’s environmental impact [8,9]. While the hazards of Cr(VI) are extensively documented, the pervasive presence of Cr(III) in environmental matrices, coupled with its potential oxidative conversion to Cr(VI), presents a compelling case for its dedicated investigation. Understanding the behavior, mobility, and effective mitigation strategies for Cr(III) addresses a critical knowledge gap in environmental engineering and chromium management practices [10]. Accordingly, this research aims to deepen the understanding of Cr(III)’s environmental dynamics and to develop more precise and efficient treatment methodologies. Such advancements align with Environmental Protection Agency (EPA) standards, which regulate total chromium concentrations in drinking water, highlighting the necessity of addressing all chromium species comprehensively [6,10].

Dedicated research in this area is still necessary for developing effective water treatment methods to mitigate the risks posed by chromium pollution. In recent years, many different techniques have been tested for Cr removal from water, which revealed both advantages and limitations. One of the most popular methods involves the use of activated carbon, which is a very effective and versatile adsorbent due to its high surface area and porous structure. Many different precursors can be used for the synthesis of activated carbons, derived from both natural materials and wastes. For instance, Behloul et al. [11] have explored innovative adsorbents, such as those derived from cotton fiber waste. However, activated carbon can be costly and may require complex agents for optimal performance [12]. Despite these drawbacks, activated carbon offers profitability, availability, and high efficiency, often reaching up to 100% effectiveness in removing heavy metals. The effectiveness of adsorption largely depends on the choice and preparation of cost-effective and eco-friendly adsorbents [13,14]. Alternatives like chitosan, alginate-based materials, and various plant wastes have been investigated for their adsorptive properties, despite some limitations like pH dependence or limited affinity for certain dyes [15,16]. Researchers like Rouibah et al. [17,18] and Thabede et al. [19] have utilized plant-based adsorbents for effective heavy metal removal, demonstrating the potential of these sustainable materials in water purification processes [20]. More recently, studies by Rasheed et al. [21], Putz et al. [22], and Coi et al. [23] have demonstrated enhanced adsorption capacities, particularly for the removal of chromium pollutants, using mesoporous silica-based inorganic sorbents. These findings underscore the importance of ongoing research efforts in identifying novel adsorbents and refining existing methodologies for effective chromium remediation.

Clay, a naturally occurring material on the earth’s surface, is composed of a blend of water, alumina, and silica along with eroded rock particles. Its abundance and cost effectiveness make it an appealing solution for environmental remediation. In particular, kaolin clay boasts a significant alumina content of 38 wt.%, with only trace amounts of other elements such as potassium oxide (K_2_O), sodium oxide (Na_2_O), and iron oxide (Fe_2_O_3_), each constituting less than 0.5 wt.%. Recognized for its adsorptive capabilities, when subjected to specialized treatment, kaolin displays an increased surface area and pore volume. This enhancement bolsters its role as an economical and potent adsorbent. Studies highlight kaolin’s ability to effectively remove a diverse array of contaminants from wastewater, including both heavy metals and organic pollutants [24,25]. Tailoring kaolin through various modifications, e.g., with phosphoric acid, has shown to heighten its selectivity and adsorption efficiency, marking its versatility in managing industrial effluents. Specific research instances, such as Mustapha et al.’s [26] work, have utilized kaolin to extract multiple pollutants from tannery effluents, demonstrating its high surface area’s contribution to pollutant removal. Similarly, El-Rabiei et al. [27] employed kaolin for the extraction of heavy metals like iron, copper, chromium, and zinc, with notable efficacy in zinc removal. Furthermore, Kaibo et al.’s [28] innovative approach to kaolin modification has shown promising results in selectively adsorbing rare earth elements from mining wastewater. These findings underscore kaolin’s potential as a customizable and effective adsorbent in wastewater treatment applications.

The current study delves into the adsorption of Cr(III) using kaolin clay sourced from the Tamazert region in Algeria. The choice of Cr(III) as the target pollutant stems from the analysis of industrial discharges from a tannery situated in the town of Jijel (Algeria), where Cr_2_O_3_ is commonly used as a tanning agent. Investigation revealed a significant presence of Cr(III) in these discharges, leading to pollution in the bay of Jijel (Algeria). Given the substantial environmental impact of Cr(III) pollution in our region, this research aims to contribute to environmental protection efforts and enhance the quality of industrial discharges. By exploring the adsorption of Cr(III) using appropriate adsorbents, we strive to develop effective remediation strategies capable of mitigating pollution caused by industrial activities, thereby safeguarding the environmental integrity of our region and promoting sustainable industrial practices.

To comprehensively understand the adsorption process, we conducted a thorough examination of the clay’s structural and textural properties using a range of analytical techniques. These techniques included X-ray diffraction (XRD), Fourier-transform infrared spectroscopy (FTIR), scanning electron microscopy coupled with energy dispersive spectroscopy (SEM-EDS), X-ray photoelectron spectroscopy (XPS), and Brunauer–Emmett–Teller (BET) surface analysis. Moreover, further aspects were investigated by laser granulometry and thermogravimetric-differential thermal analysis (TGA–DTA) alongside the assessment of the point of zero charge (PZC) and zeta potential. This comprehensive characterization was pivotal in determining the adsorption equilibrium and delineating the optimal conditions for maximum Cr(III) removal. In addition, by integrating density functional theory (DFT) with the atoms in molecules framework, we offer a novel approach to decode the adsorption process at both the macroscopic and molecular levels. The synergy of experimental procedures with advanced computational techniques such as the conductor-like screening model for real solvents (COSMO-RS), independent gradient model (IGM), reduced density gradient (RDG), and quantum theory of atoms in molecules (QTAIM) analyses also enabled us to unravel the complex interaction mechanisms between Cr(III) ions and kaolin, providing deep insights into the nature and strength of intermolecular forces.

## 2. Results and Discussions

### 2.1. XRD and FTIR Characterization

**Figure 1:**
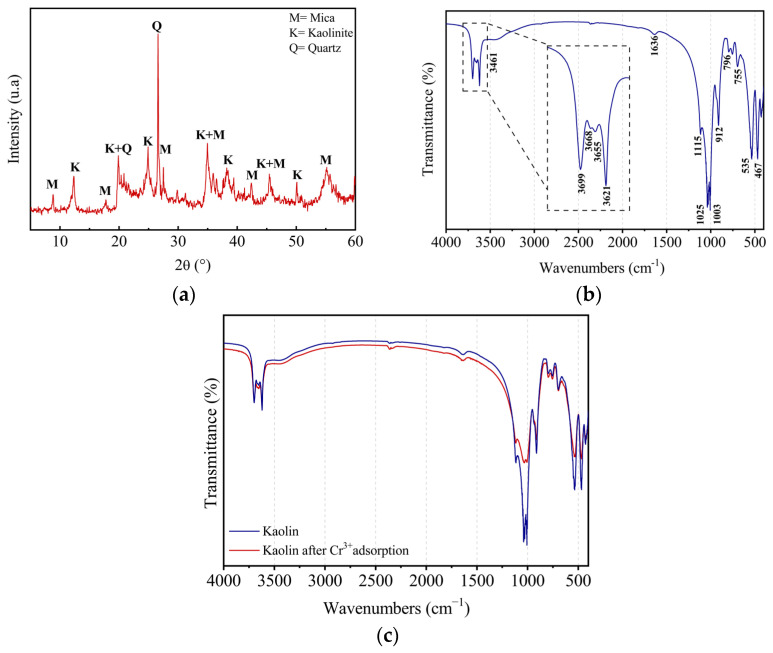
Structural characterization of kaolin: X-ray diffraction patterns of kaolin (**a**) and FTIR spectrum of kaolin after and before Cr^3+^ adsorption (**b**,**c**).

The recorded XRD diffractogram presented in Figure 1a reveals the presence of three crystalline phases: (1) kaolinite (JCPDS PDF 14-0164) Al_2_O_3_, 2SiO_2_, 2H_2_O, or Si_2_Al_2_O_5_OH_4_; (2) quartz SiO_2_ (JCPDS PDF 46-1045); and (3) muscovite(mica) (JCPDS PDF 07-0032). Muscovite is a silicate clay mineral with the chemical formula KAl_2_(OH, F)_2_ [AlSiO_3_O_10_]. Table 1 presents the d_hkl_ values of the three phases identified for the main characteristic peaks.

The IR spectrum of kaolin (Figure 1b) confirms the predominance of the kaolinite phase. Four OH-stretching vibration modes typical of well-crystallized kaolinite were observed [29,30]. The band located at 3621 cm^−1^ corresponds to the internal OH groups, located between the octahedral and tetrahedral sheets, while those located at 3699, 3668, and 3655 cm^−1^ are linked to external OH groups [31]. The valence vibrations of adsorbed water molecules are represented by the existence of a tiny band at 3461 cm^−1^. At 1636 cm^−1^, the deformation peak corresponding to the HOH bonds is found. Si–O bond-stretching vibrations are manifested by the intense peaks at 1115 cm^−1^ (in longitudinal mode), 1003, and 1025 cm^−1^ (in plane). The peak at 1025 cm^−1^ is characteristic of the Si–O bonds of muscovite [32], which is in perfect coherence with the results of the XRD analysis. Si–O–Al bonds are characterized by the existence of two bands at 796 and 755 cm^−1^. The peak detected at 912 cm^−1^ is characteristic of the vibrational deformation of the OH groups inside the Al–OH bonds. A peak at 535 cm^−1^ attributed to Al–O–Si (Al octahedron) deformation vibrations and another at 467 cm^−1^ attributed to Si–O–Si bond deformation vibrations was also observed.

Finally, there are no notable bands in the IR spectrum that correspond to organic matter. In addition, the FTIR analysis was further examined after chromium adsorption. As shown in Figure 1c, the location of the peaks after adsorption did not change; however, the peak intensities decreased, which confirms an ion exchange between Cr^3+^ and the OH group and surface complexation between Cr^3+^ and Al and Si on the surface of the clay [33].

### 2.2. SEM-EDS Characterization

**Figure 2:**
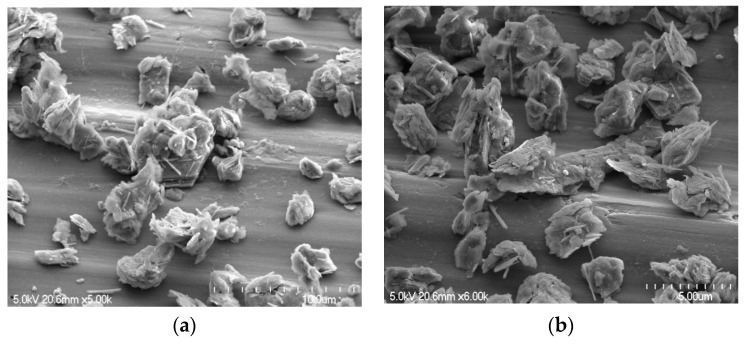

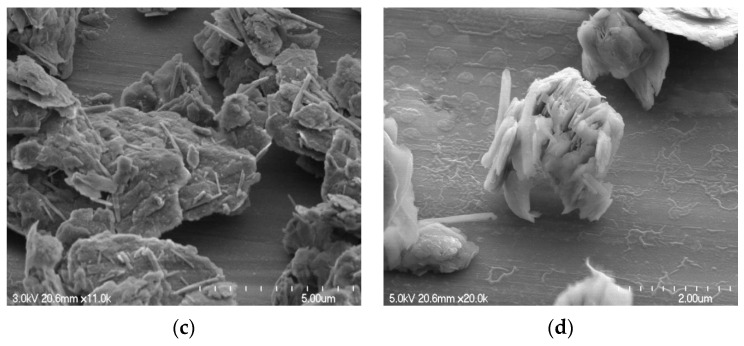
SEM images of kaolin at magnifications ×10.00 (**a**), ×5.00 (**b**,**c**), and ×2.00 µm (**d**).

**Figure 3:**
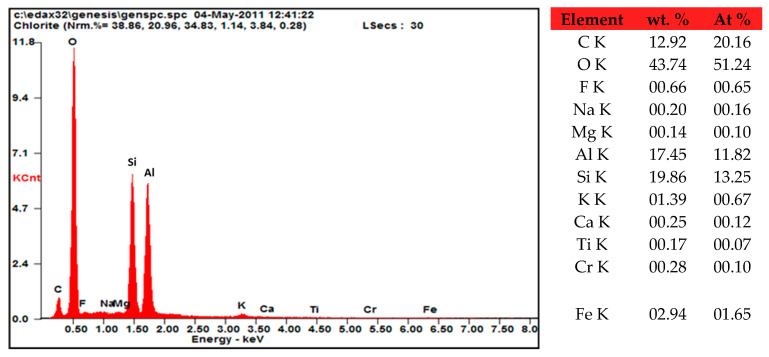
Kaolin EDS microanalysis.

Figure 2 depicts the morphology of the Tamazert kaolin. Kaolin is composed of compacted clusters taking the form of sheets and needles of micrometric sizes (from 1 to 9 µm); the laminated structure is a characteristic of well-crystallized kaolinite. However, the texture study by SEM with the practiced enlargement did not allow us to highlight the porous structure of the support. Elemental composition of the kaolin, as determined by EDS spectroscopy, is depicted in Figure 3. Additionally, the ceramics unit of El Milia Company conducted a comprehensive chemical analysis of the kaolin using X-ray fluorescence (XRF), the results of which are presented in Table 2. This analysis revealed a high content of SiO_2_ and Al_2_O_3_, underscoring their dominance in the kaolin’s composition, as referenced in [34]. These results are consistent with the EDX analysis, which showed a Si/Al mass ratio greater than one. This demonstrated that kaolinite (Al_2_O_3_, 2SiO_2_, 2H_2_O), an essential mineral of kaolin, was predominant. It also indicated the presence of silicas in the form of quartz or phyllosilicates of type (2:1), and the XRD analysis revealed that it is muscovite. EDS measurement also revealed the presence of carbon at a significant atomic percentage. The presence of carbon can be partly linked to the organic compounds contained in clay soil. The ignition loss of KT2 kaolin was 10.5% (pure kaolin weight loss = 13.95%), which can explain the existence of organic compounds in the material. In addition, we found that our support contained iron and traces of chromium.

### 2.3. Laser Diffraction Particle Size Analysis

**Figure 4:**
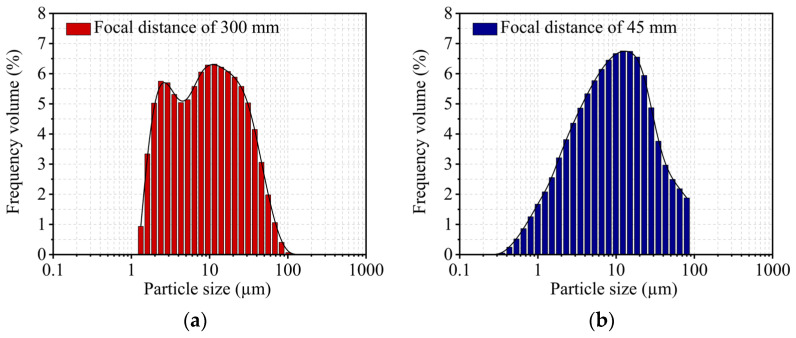
Particle size distribution of kaolin: (**a**) focal distance of 300 mm and (**b**) focal distance of 45 mm.

Figure 4 displays the results of the kaolin particle size analysis, and particle diameters ranged from 1.16 to 100 µm. According to the particle size distribution curve, two populations could be identified based on their particle diameters of 2.8 and 10 µm (bimodal distribution with a focal length of 300 mm). These outcomes line up with SEM observations of the support texture. A decreased focus distance resulted in a different form of the particle size curve, which indicated the presence of particles between 0.35 and 1 µm in size. Table 3 lists the particle size quantities, including the volume diameters D_10_ and D_90_, the median diameter D_50_, and the Sauter average D (3.2).

### 2.4. XPS Analysis

**Figure 5:**
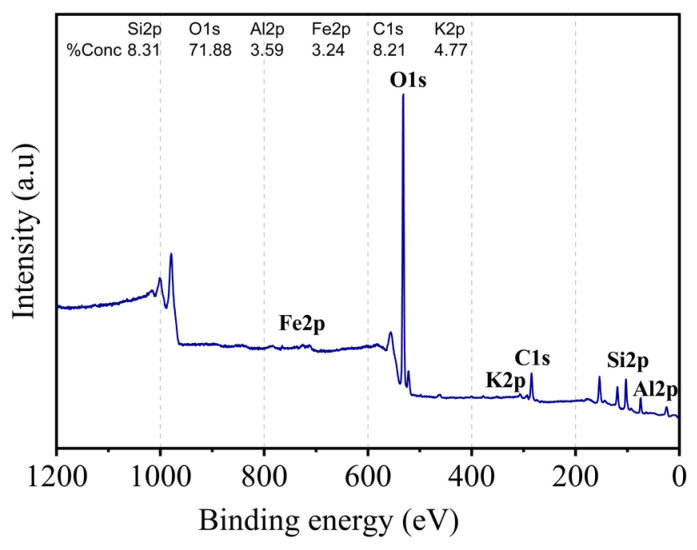
XPS survey spectra of kaolin.

**Figure 6:**
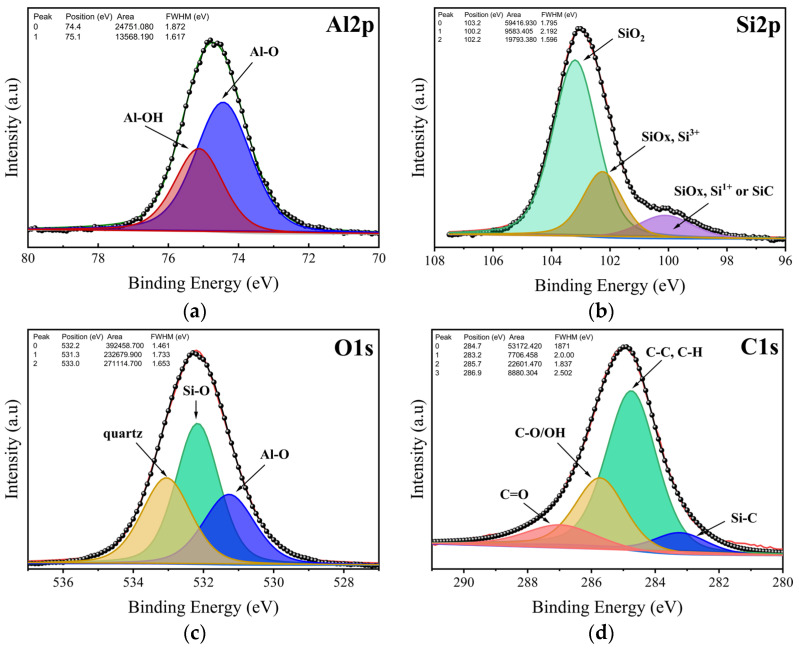
High resolution of XPS spectra of (**a**) Al 2p, (**b**) Si 2p, (**c**) O 1s, and (**d**) C 1s.

The surface composition of kaolin was obtained by XPS analysis. The survey of kaolin is given in Figure 5, whereas the binding energy and atomic % of the elements identified are reported in Table 4. Silicon, oxygen (which had the largest concentration), aluminum, iron, and potassium were the constituent elements of the kaolin surface. The presence of carbon was also revealed, which is consistent with the EDS study. The carbon content indicates the rate of organic matter in the examined surface layer. The Al 2p, Si 2p, O 1s, as well as C 1s peaks were subjected to additional fitting procedures to shed light on the possible bonds in the studied kaolin sample, as illustrated in Figure 6. The Al 2p photoelectron line consisted of two peaks situated at 74.4 eV and 75.1 eV (Figure 6a). They could be associated with Al–O and Al–OH bonds. In the spectra of Si 2p (Figure 6b), three peaks were found at 103.2 eV, 102.2 eV, and 100.2 eV. The peak at 102.2 eV belonged to kaolinite, while the one at 103.2 eV confirmed the presence of quartz. The third peak with the lowest intensity could be associated with the existence of a small amount of SiOx or SiC formation. The O 1s line was resolved into three contributions attributed to Al–O and Al–OH bonds at about 531.3 eV, Si–O bonds at 532.2 eV, and quartz at about 533 eV (Figure 6c). All of this is in agreement with the previously reported results for kaolinite and similar samples [35].

Finally, we also focused on the C 1s peak, which displayed a complex nature. The outcome of the fit is illustrated in Figure 6d. Within this spectrum, we successfully identified Si–C, C–C/C–H, C–O, and C=O bonds. Specifically, the peak associated with C–C and C–H bonds represented adventitious carbon, originating from surface impurities of hydrocarbons, as detected in all samples during XPS analysis.

### 2.5. Specific Surface Area and Porous Structure

**Figure 7:**
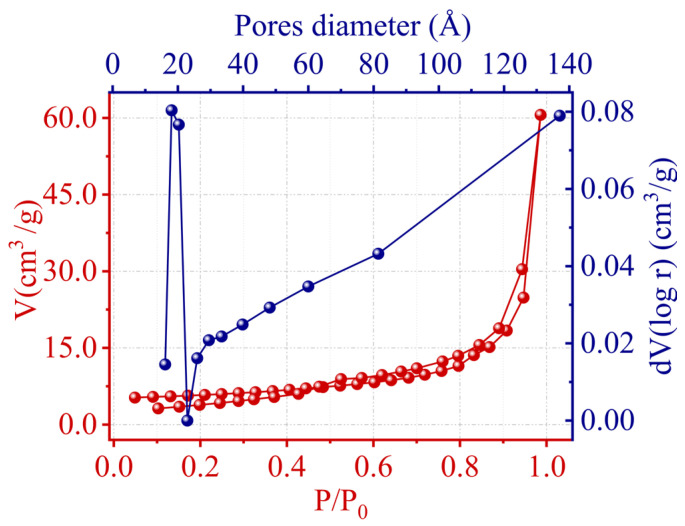
N_2_ adsorption–desorption and pore size distribution.

The specific surface area, total pore volume, and average pore diameter of kaolin were determined using nitrogen gas adsorption–desorption isotherms analyzed by the BET method. These textural properties are summarized in Table 5, with Figure 7 depicting the corresponding pore size distribution. From the BET analysis, the specific surface area was found to be 18.376 m^2^/g, and the total pore volume was measured at 0.09376 cm^3^/g. The average pore diameter was 20.4 nm, suggesting a predominance of larger mesopores. Figure 7 provides further insight, showing a bimodal pore size distribution with a marked peak at approximately 2 nm, which corresponded to a mesopore-rich population, and evidence of micropores under 2 nm in diameter. This bimodal distribution is significant, as it suggests that kaolin possesses a hierarchical porosity that could be advantageous for various adsorption processes, including the adsorption of Cr(III) ions from aqueous solutions.

### 2.6. TGA-DTA

**Figure 8:**
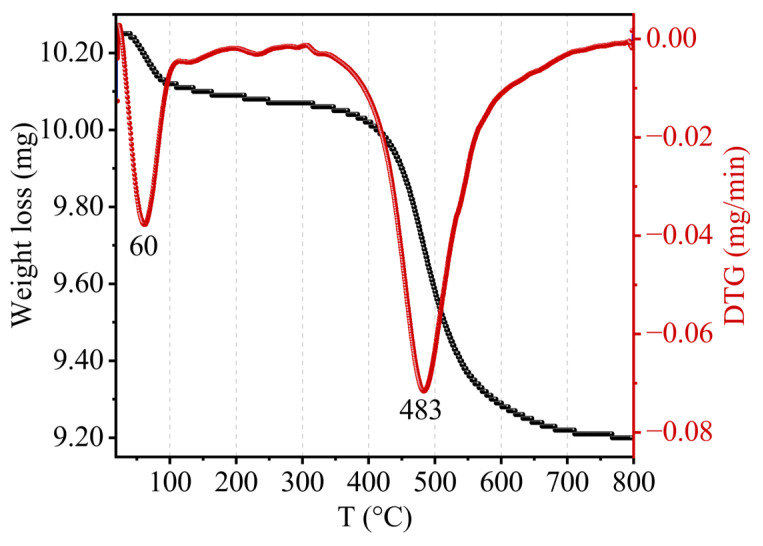
TGA–DTA curves of kaolin.

Kaolin’s TGA–DTA thermal study clearly demonstrated that mass loss occurred in two stages, as presented in Figure 8. The first stage corresponded to a mass loss of 0.58% at low temperatures (about 60 °C) due to hygroscopic water desorption and demonstrated the hydrophilic character of clay. The second mass loss, approximately 9.56%, occurred around [400–600 °C] and was proportional to the loss of OH ions from the structure. This corresponded to the loss of the constituent water (crystalline water) by a diffusion process as well as the transformation of kaolin into metakaolin via the following reaction:

(1) $${Al}_{2}O_{3},\;2SiO_{2},\;2H_{2}O\to {Al}_{2}O_{3},\;2SiO_{2}+2H_{2}O_{vap}$$

The dehydroxylation of kaolin took place at a lower temperature than that of pure kaolinite (515 °C), which suggests the presence of a significant amount of adsorbed water in the interlayer space of kaolinite.

### 2.7. pH_PZC_ Determination

**Figure 9:**
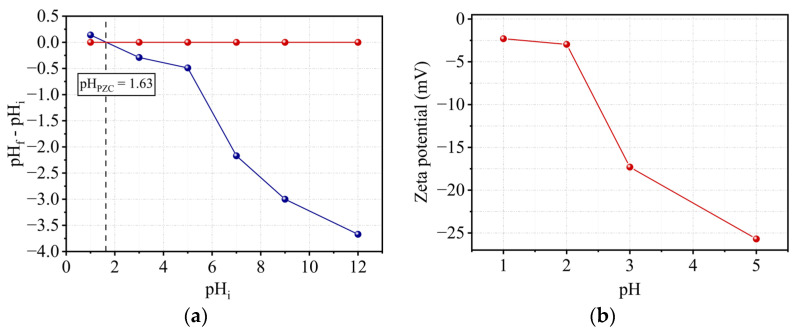
(**a**) Kaolin point of zero charge measurement and (**b**) zeta potential values of kaolin as a function of pH.

The pH of a solution in equilibrium with a material whose total surface charge is zero is defined as the point of zero charge (PZC). It might reflect the complete absence of a charge or the precise compensation of positive and negative charges. The zero charge point of kaolin was discovered to be 1.63 (Figure 9a). Thus, for pH values less than pH_PZC_, by consuming the solution’s protons, the surface of the support becomes positively charged, resulting in a less acidic solution. For higher values, the surface becomes negatively charged as a result of the release of these protons, resulting in a more acidic solution.

### 2.8. Zeta Potential

A particle’s zeta potential is an estimate of the global charge that the particle obtains in the liquid medium. As a result, the stability of emulsion and suspension dispersions is heavily dependent on this potential, the magnitude of which gives an indication of the repulsion force present and allows for the prediction of particle stability. A suspension with a zeta potential lower than + (or −) 10 mV is often unstable, and the particles tend to gather and flocculate. A value greater than + (or −) 20 mV indicates the relative stability of the particles [36], which repel each other and cannot stick together, while a value greater than + (or −) 30 mV gives them good stability.

More broadly, it is widely believed that the higher the zeta potential, the more stable the emulsion particles become, and the lower the zeta potential, the closer they are to particle aggregation. Figure 9b illustrates the findings of this investigation. Unlike the other physico-chemical characteristics (specific surface area, size, shape, formula), the zeta potential is not fixed. It is unique in that it varies depending on the particle’s environment. The pH of the medium, as demonstrated in our work, is a significant component that can alter the zeta potential of the particles. When the pH rises, the zeta potential of the support drops. The surface charge explains these values for kaolin. The latter was observed on the layer side faces (positive, negative, or neutral charge) and the basal surface (always negative). The basal and lateral surfaces were about identical in size. When the pH was reduced, protonation of the OH carried on the lateral surfaces reduced the negative charge, and thus the zeta potential decreased. The best stability was obtained at pH 5.

### 2.9. Chromium Adsorption Study

#### 2.9.1. Effects of Contact Time and Initial Concentration

**Figure 10:**
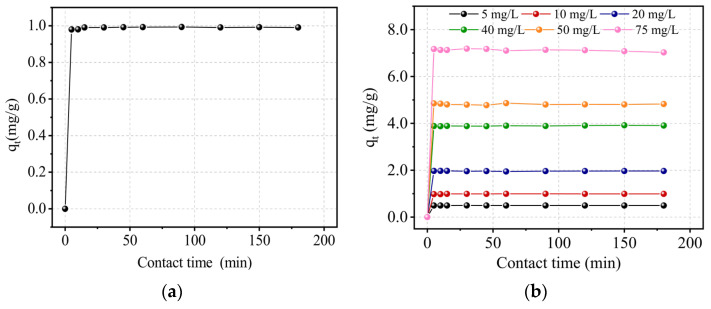
Effect of the contact time (**a**) and initial concentration (**b**) on the adsorption capacity of the adsorbent under constant conditions: pH = 5, T = 22 °C, V = 300 rpm, and r = 10 g/L.

Figure 10a depicts the influence of contact time on the Cr(III) adsorption on kaolin at a concentration of 10 mg/L. After 15 min, equilibrium was reached, and 99.11% of the chromium was adsorbed. This finding demonstrates kaolin’s high affinity for Cr(III). The influence of the initial concentration of Cr(III) for concentrations of 5, 20, 40, 50, and 75 mg/L was investigated under the same conditions, also showing the great affinity of Kaolin for the cation (Figure 10b). Indeed, increasing the concentration of Cr(III) reduced the retention rate, but adjusting the concentration from 5 to 50 mg/L kept it within the range [97–100%]. However, at a higher concentration of 75 mg/L, it reached 95%. Furthermore, for the various concentrations examined, adsorption was found to be relatively rapid.

#### 2.9.2. Effect of pH

**Figure 11:**
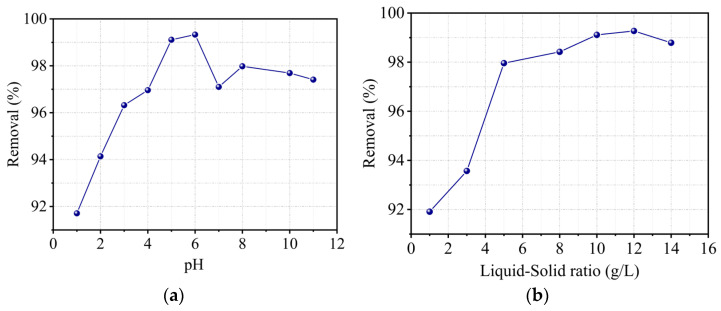
Effect of (**a**) pH and (**b**) solid–liquid ratio on the removal percentage of Cr(III) under constant conditions: C_0_ = 10 mg/L, T = 22 °C, t_c_ = 120 min, V = 300 rpm, and r = 10 g/L.

Figure 11a illustrates the effect of solution pH, which demonstrates that increasing the pH from 1 to 6 enhances the Cr(III) adsorption capacity, but a further increase determines a non-monotonous decline, as previously established by many authors. For instance, Dakiky et al. [37] investigated Cr(III) adsorption on various supports and determined that maximum adsorption occurs at a pH of 5. Activated carbon extracted from agricultural waste in several studies, such as Fahim et al. [38], and from industrial waste, such as the one investigated by Mohan et al. [39], showed similar results. The rise in adsorption removal in the pH range 1–6 could be explained on the one hand by an increase in the clay’s cation exchange capacity as a result of Si–OH and Al–OH groups hydrolysis [40] or by a decrease in the occurrence of competition between the H^+^ protons and the Cr^3+^ cation on the negatively charged support’s adsorption sites. Rivera-Utrilla and Sanchez-Polo [41] investigated the distribution of Cr(III) species in an aqueous solution as a function of pH and obtained the following results:

- At pH 2, chromium is found only in its cationic form Cr^3+^ (hexahydrate).
- At pH 4, the predominant species are Cr^3+^ (61.16%) and Cr(OH)^2+^ (38.60%).
- At pH 6, chromium is found in the form of Cr(OH)^2+^ (60.61%), Cr(OH)^2+^ (38.24%), Cr^3+^ (0.96%), and Cr(OH)_3_ (0.19%).
- For pH values higher than 7, Cr(OH)_3_ and Cr(OH)^4−^ species emerged, and the adsorption capacity decreased.

At last, by varying the pH, the following equations [41,42] are taken into account:

(2) $$SiOH+{Cr}^{3+}\to SiO{Cr}^{2+}+H^{+}$$

(3) $$SiOH+{Cr}^{3+}+{OH}^{-}\to SiO{Cr}^{+}+H_{2}O$$

(4) $$SiOH+{Cr}^{3+}+{2OH}^{-}\to SiO{Cr}^{+}{\left(OH\right)}_{2}$$

(5) $$SiOH+{Cr}^{3+}+{4OH}^{-}\to SiO{Cr}^{+}{\left(OH\right)}_{4}^{4+}$$

The transition from the formation of SiOCr^2+^ species in a relatively acidic medium to SiOCr^+^, then $SiO{Cr}^{+}{\left(OH\right)}_{2}$, and finally $SiO{Cr}^{+}{\left(OH\right)}_{4}^{4+}$ in a relatively neutral medium can thus be noted. In this study, the optimal pH was set at 5, since the zeta potential study showed that the best stability of kaolin particles was obtained at this pH value.

#### 2.9.3. Effect of Solid–Liquid Ratio

The results of the effect of this parameter, illustrated in Figure 11b, show on the one hand that kaolin is a very effective adsorbent for trivalent Cr and on the other hand that adsorption removal increases with adsorbent concentration. Indeed, the percentage of removal increases from 91.91% for a ratio of 1 to 97.96% for a ratio of 5 (g/L) and reaches 99% for higher values. As previously stated, this phenomenon is explained by an increase in the amount of accessible adsorption sites on the surface of the support.

#### 2.9.4. Effect of Ionic Strength

**Figure 12:**
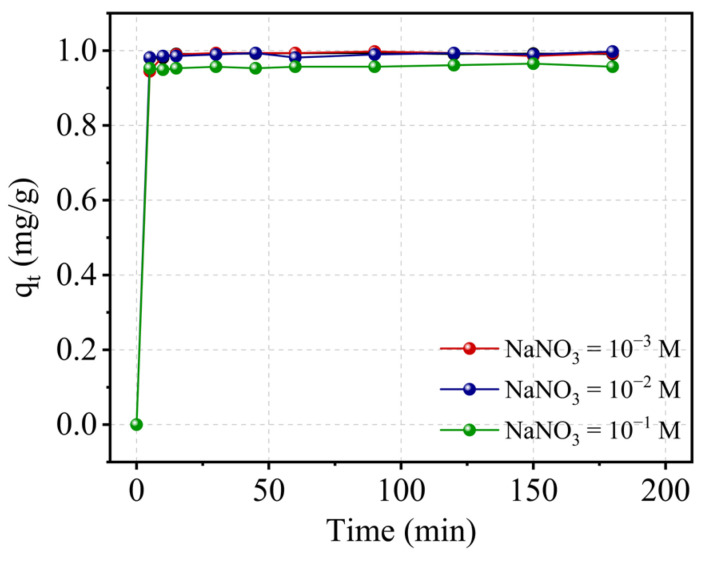
Ionic strength’s effect on the adsorption capacity of adsorbent under constant conditions: pH = 5, T = 22 °C, V = 300 rpm, and r = 10 g/L.

In general, the influence of ionic strength is defined by the nature of the adsorbate, the adsorbent as well as the chemical agents used (NaCl, KCl, NaNO_3_, CaCl_2_, and so on) and their concentrations. As a result, ionic strength can either reduce or increase the rate of adsorption. At an initial pH of 5, different quantities of NaNO_3_ nitrate were added to the system to investigate the effect of ionic strength on the Cr(III) adsorption on kaolin. The obtained findings are shown in Figure 12. The graphs for 10^−3^ and 10^−2^ M concentrations are precisely superimposed on the kaolin alone, demonstrating that the addition of NaNO_3_ at low concentrations did not affect the adsorption process. By raising the salt concentration, the adsorption rate was reduced by 5% within the first few minutes. Changes in the pH of the suspended particles, which impact the diffuse layer, are commonly linked to this inhibition [43]. The pH of the solution decreased as the concentration of the salt increased, which explains, on the one hand, the decrease in adsorption capacity. Based on the double layer theory, increasing the ionic strength for pH > pH_PZC_ values reduces the potential of the diffuse layer and the forces of attraction between the surface and the specifically adsorbed ions [44,45,46,47]. As a result, the thickness of the double layer that surrounds the kaolin particles is assumed to have decreased as the ionic strength increased, and thus the adsorption rate was reduced.

### 2.10. Effect of Temperature and Thermodynamic Studies

**Figure 13:**
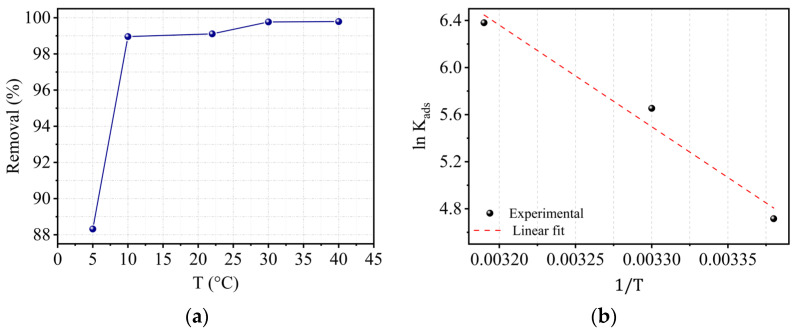
(**a**) Temperature’s effect for the conditions C_0_ = 10 mg/L, pH = 5, V = 300 rpm, and r = 10 g/L and (**b**) variation of the adsorption constant of Cr(III) as a function of temperature.

The thermodynamic parameters ∆G^0^, ∆H^0^, and ∆S^0^ of the Cr(III) adsorption equilibrium on kaolin were calculated by Equations (6) and (7). Figure 13a illustrates the temperature’s effect on adsorption, and Figure 13b shows the variation of the adsorption constant as a function of temperature. The calculated parameters are listed in Table 6.

(6) $$∆G^{0}=-RT\ln k_{ads}$$

(7) $$\ln k_{ads}=\frac{∆S^{0}}{R}-\frac{∆H^{0}}{RT}$$

The retrieved results allowed drawing the following conclusions:

- The negative values of ∆G^0^ show that the adsorption of Cr(III) on Tamazer kaolin is spontaneous.
- As the temperature gets higher, the values of ∆G^0^ become more negative, indicating that adsorption is more favorable at higher temperatures. Furthermore, the positive value of the enthalpy proves that the adsorption is endothermic and also favored by the rising temperature. In fact, several studies have demonstrated that the Cr(III) adsorption on kaolinite is endothermic [45,48].
- The high value of the enthalpy suggests the occurrence of chemical adsorption. However, it is reported in the literature that if ∆G^0^ values are below 18 kJ·mol^−1^ (absolute value), the adsorption process is dominated by a physisorption [48]. Thus, the adsorption of Cr(III) may be interpreted as physisorption complemented by chemisorption on the kaolin surface.
- The increase in the degree of freedom of the adsorbed species, as well as the rise in disorder at the solid–adsorbate interface, explains why entropy is positive.

The activation energy (*E_a_*) is a crucial thermodynamic quantity that can be empirically measured by computing the kinetic rate constants at different temperatures. The empirical law of Arrhenius is represented in terms of the kinetic constant *k*:

(8) $$k=A\exp\left(\frac{-E_{a}}{RT}\right)$$

where *A* is a pre-exponential or frequency factor, *R* is the perfect gas constant (*R* = 8.314 J·mol^−1^·K^−1^), *T* is the absolute reaction temperature, and *E_a_* is the activation energy. The plot ln*k*_2_ vs. (1/*T*) allows for calculating the activation energy *E_a_*. The activation energy value reveals the nature of the phenomenon. A value of *E_a_* = 25.665 kJ·mol^−1^ was obtained, indicating a predominant chemical adsorption phenomenon [49]. When the value of *E_a_* < 40 kJ·mol^−1^, the process is generally controlled by the diffusion phenomenon, whereas values of *E_a_* > 40 kJ·mol^−1^ imply that the adsorption is governed by a chemical process. This suggests that the adsorption of Cr(III) on kaolin is governed by a diffusion phenomenon in the pores.

### 2.11. Adsorption Isotherm Study

**Figure 14:**
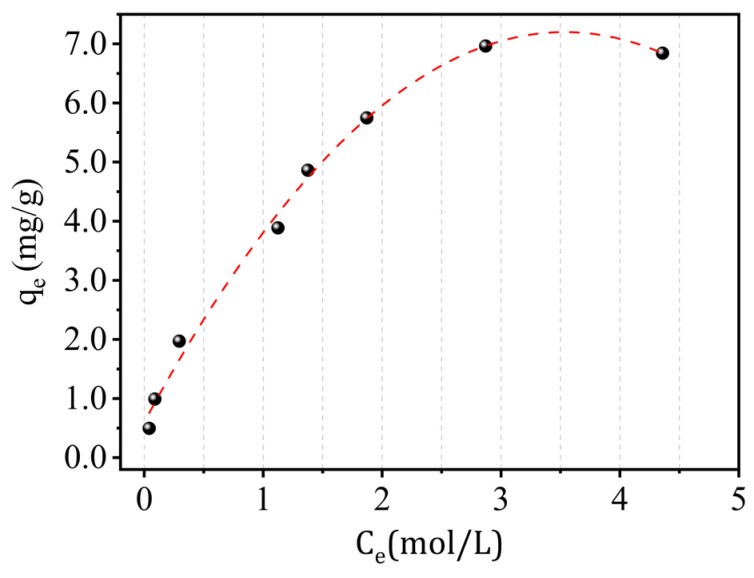
Adsorption isotherm of Cr(III) on kaolin with the conditions V = 300 rpm, pH = 5, T = 22 °C, and r = 10 g/L.

Figure 14 represents the isotherm of adsorption of Cr(III) on kaolin at an ambient temperature (*T* = 22 °C) by a classic form of Langmuir isotherm of type (L) having the highest possible adsorption capacity of 7.10 mg/g. The adsorption isotherm of Cr(III) on kaolin was analyzed using a variety of models, including Langmuir, Freundlich, Temkin, Elovich, and Dubinin–Radushkevitch. All the calculations carried out for the various models tested are given in Table 7.

- Langmuir’s model gives a good representation of the Cr(III) isotherm (*R*^2^ > 0.98). The applicability of both of these versions suggests good adsorption at low concentrations.
- The separation factor *R_L_* ranges between 0.06 and 0.14, indicating that adsorption is favorable. The value of *R_L_* is given by the equation:

(9) $$R_{L}=\frac{1}{\left(1+bC_{e}\right)}$$

Table 8 provides a comprehensive compilation of studies investigating the removal of Cr(III) using different adsorbents. The data presented in the table demonstrate that the equilibrium data were effectively fitted to the Langmuir isotherm model, underscoring its applicability to describe the adsorption behavior of chromium across various adsorbent materials.

### 2.12. Computational Study

#### 2.12.1. Structural and Optimization Analysis

**Figure 15:**
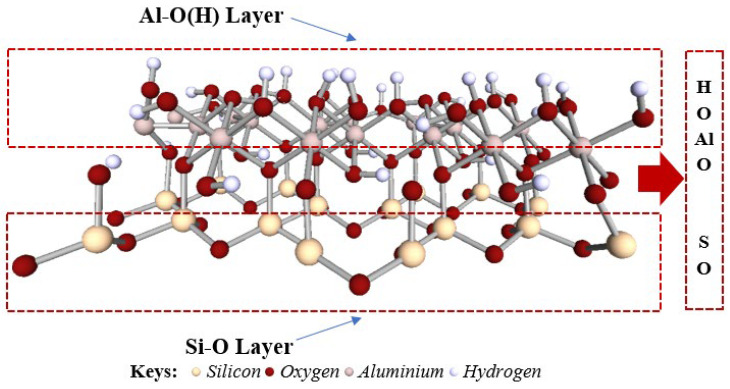
Detailed description with side and top views for the structure model of the kaolinite surface.

The detailed structural model of the investigated kaolinite surface is shown in Figure 15, showcasing the distinct Al–O(H) and Si–O layers. The Al–O(H) layer is characterized by a dense arrangement of hydroxyl groups attached to aluminum atoms, a configuration that facilitates hydrogen bonding and contributes to the surface’s chemical reactivity. On the other hand, the Si–O layer consists of a network of silicon-oxygen tetrahedra, presenting a more ordered and less reactive surface [54]. These structural characteristics are essential for understanding the surface chemistry of kaolinite, particularly how it interacts with various adsorbates, including Cr(OH)_3_. This structural assessment, complete with multi-angled visualizations, is critical for advancing adsorption studies and informs subsequent COSMO-RS charge surface analyses, facilitating a deeper comprehension of the interaction intricacies between kaolinite and adsorbed species.

The COSMO-RS model facilitates an in-depth examination of the electrostatic mapping of the kaolinite surface in the presence of Cr(OH)_3_, with varying colors representing different potential interactions, as displayed in Figure 15.

**Figure 16:**
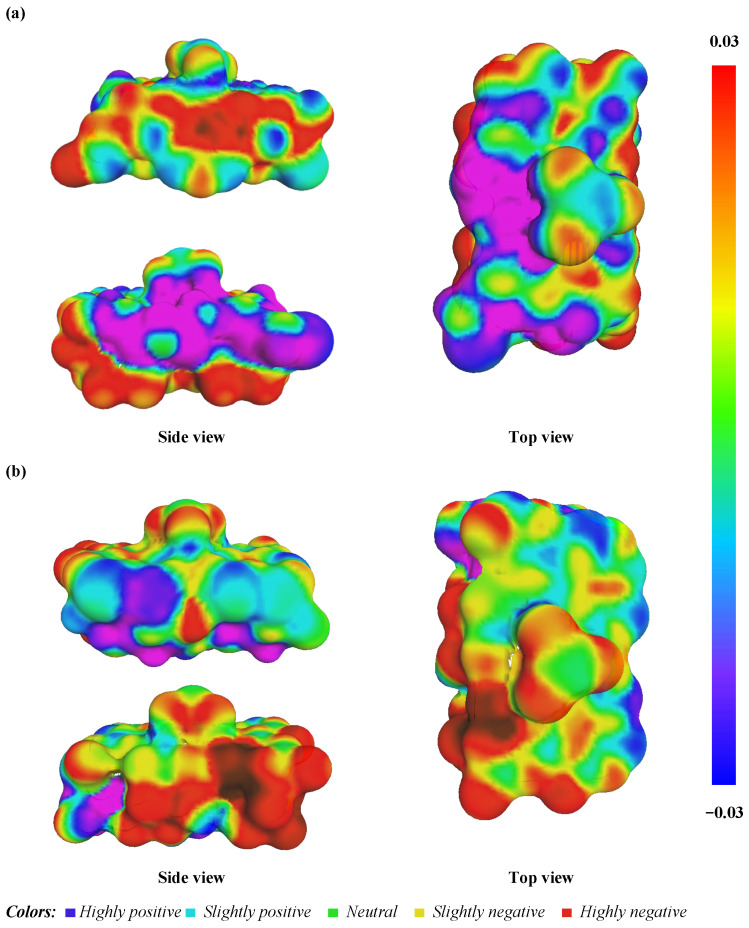
Detailed description of the COSMO-RS distribution with side and top views for the adsorbed Cr(OH)_3_ onto kaolinite surface in Al–O(H) (**a**) and Si–O (**b**) layers.

The Al–O(H) layer’s electrostatic profile is rich with both hydrogen bond donor (blue) and acceptor (red) sites, interspersed with non-polar (green) regions (Figure 16a). This intricate pattern suggests a highly interactive surface capable of forming diverse non-covalent interactions with Cr(OH)_3_. Such a landscape is compatible with both physisorption, due to the non-polar regions, and chemisorption, facilitated by the polar sites. By comparison, the Si–O layer (Figure 16b) displays a more homogeneous green profile indicative of a primarily physisorptive interaction potential due to its non-polar character, which is in accordance with the experimental results reported in the thermodynamic study. The limited red and blue areas imply fewer sites for strong directional bonding, such as hydrogen bonds, which may lead to a weaker interaction with Cr(OH)_3_. The contrasting electrostatic environments of the Al–O(H) and Si–O layers align with the hypothesis that the Al–O(H) layer would exhibit a higher adsorption capacity and specificity for Cr(OH)_3_. This is due to its ability to engage in multiple types of interactions, potentially leading to a more stable and selective adsorption process. These findings are significant for the engineering of kaolinite-based adsorbents, where maximizing the adsorption capacity and selectivity for specific contaminants is crucial. Understanding these surface interactions at a molecular level enables the design of targeted adsorption processes and the development of kaolinite materials with optimized properties for the removal of Cr(III) from aqueous environments.

#### 2.12.2. Molecular Orbital Analysis

**Figure 17:**
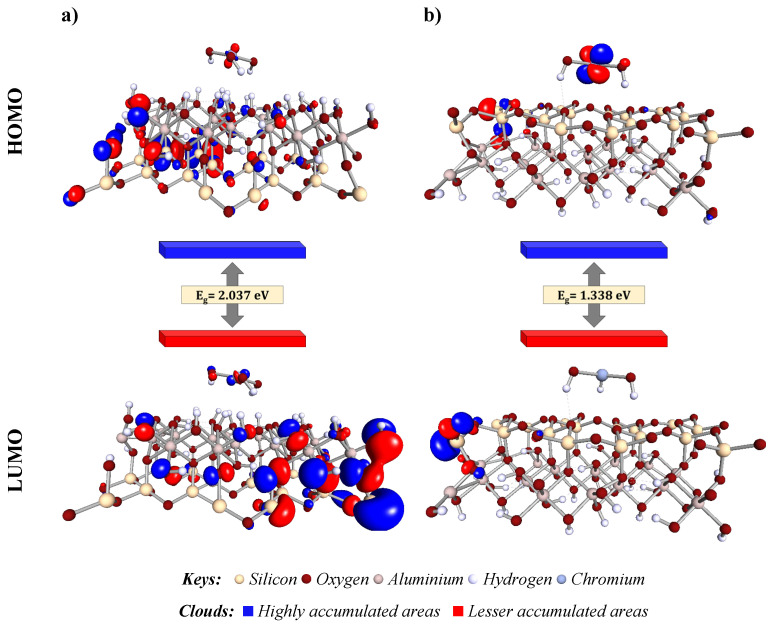
Frontier orbital distributions of the structure models for the adsorbed Cr(OH)_3_ onto kaolinite surface in Al–O(H) (**a**) and Si–O (**b**) layers.

In this study, we conducted an in-depth molecular orbital analysis to elucidate the electronic interactions that facilitate the adsorption of Cr(OH)_3_ onto kaolinite, specifically targeting the Al–O(H) and Si–O layers. The frontier orbitals, namely the HOMO and the LUMO orbitals, were critically assessed (refer to Figure 17). These orbitals are quintessential to predicting the chemical reactivity and interaction dynamics of the adsorbate–adsorbent pairs, as they define the pathways for electronic transitions essential in adsorption phenomena [16,55]. Figure 16 demonstrates that the Al–O(H) layer exhibited a band gap of 2.037 eV, suggesting a higher resistance to electron transition compared to the Si–O layer’s band gap of 1.338 eV. These band gaps reflect the reactivity potential of each layer, with a smaller gap in the Si–O layer indicating a predisposition for electron movement and potential for stronger chemical reactions. The HOMO is densely concentrated around the Cr(OH)_3_ and the Al–O(H) layers, indicating a high likelihood for electron donation. In contrast, the HOMO’s presence is reduced on the Si–O layer, pointing to weaker electronic interactions. The LUMO distribution complements this, with the Al–O(H) layer showing a propensity to accept electrons, whereas the Si–O layer’s LUMO is closely associated with the silicate matrix, suggesting different adsorption behaviors [54,56].

These findings contribute to a detailed understanding of the electronic interactions that dictate adsorption efficacy. A direct comparison with literature values highlights the distinct electronic environment due to Cr(OH)_3_, aiding in designing tailored kaolinite for specific adsorption tasks [57]. This molecular orbital analysis underscores the enhanced reactivity of the Al–O(H) layer for Cr(OH)_3_ adsorption over the Si–O layer and suggests strategies for improving adsorption processes for Cr(III) removal, enhancing kaolinite’s role in wastewater treatment.

#### 2.12.3. Non-Covalent Interaction Analysis

The elucidation of adsorption mechanisms through the visualization of weak interactions between Cr(OH)_3_ contaminant and kaolinite adsorbents is a pioneering approach. In this investigation, the IGM and RDG analyses via the Multiwfn software 3.8 packages [58] were employed to clarify the non-covalent forces involved.

**Figure 18:**
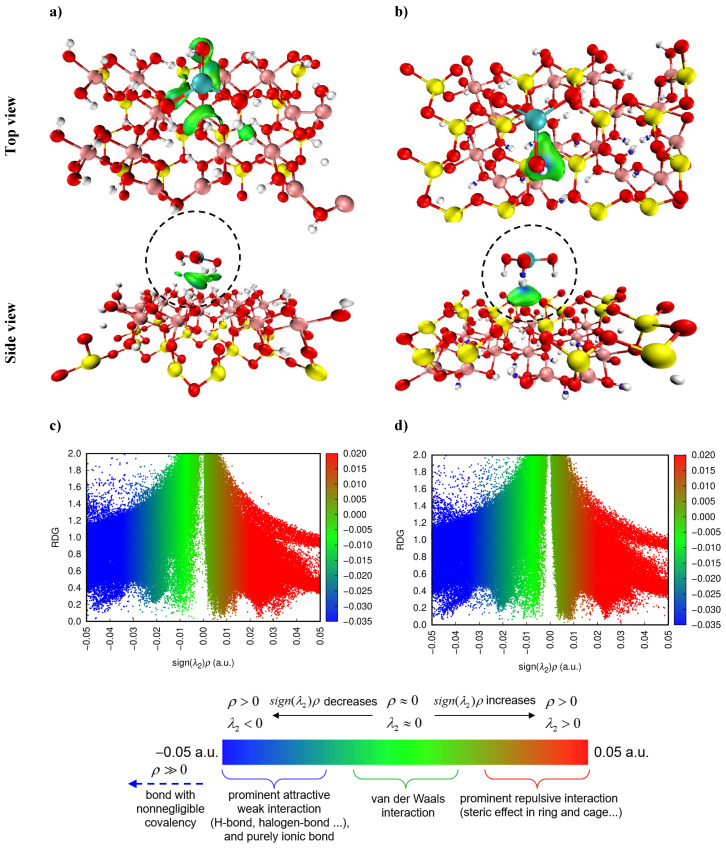
The visualized IGM weak interaction regions (isosurface value = 0.05 a.u.) and the RDG scatter map of the kaolinite surface attached to Cr(OH)_3_ in Al–O(H) (**a**,**c**) and Si–O (**b**,**d**) layers.

Employing IGM, predicated on promolecular density, we explored both interfragment (δg inter) and intrafragment (δg intra) interactions [56,58]. This study focuses on δg inter to illuminate the interaction landscapes between Cr(OH)_3_ and the kaolinite surface, modeled to account for both Al–O(H) and Si–O layers’ attachment scenario. The color-coded isosurface transitions from blue to green to red, corresponding to the intensity of attractive interactions. For instance, as depicted in Figure 18a,b, the substantial hydrogen bonding interactions indicated by the pronounced blue regions in the IGM isosurface can be correlated with the dense localization of the HOMO around the Cr(OH)_3_ molecule, particularly in the Al–O(H) layer (Figure 18a). This suggests that the electronic structure of the Cr(OH)_3_-kaolinite complex, as revealed by the molecular orbitals, is conducive to strong hydrogen bonding [59,60,61,62].

Moreover, the RDG analysis, which highlights significant electron density peaks and therefore strong hydrogen bonding at the Al–O(H) interface, aligns with the higher band gap energy observed for this layer. This implies that while the electron transfer may be less frequent due to the higher energy barrier, the interactions that do occur are likely to be more stable, as evidenced by the strong hydrogen bonds [63]. In particular, RDG analysis, showcasing notable electron density peaks at the Al–O(H) interface with Cr(OH)_3_, aligns with the higher band gap energy of this layer. This implies that, while electron transfer may be less frequent due to the increased energy barrier, the interactions that do occur tend to be more stable, as evidenced by the significant hydrogen bonding. The observed peak in electron density at sign(λ_2_)ρ values around −0.04 and −0.05 a.u. (Figure 18d) indicates robust attractive interactions, likely hydrogen bonds, more intense than those at the Si–O sites.

This finding not only validates the presence of substantial bonding interactions but also highlights the differing adsorption capacities between the Al–O(H) and Si–O layers in kaolinite. Such insights are pivotal for comprehending the adsorption mechanism and tailoring adsorbents effectively. Consequently, IGM and RDG analyses present a detailed portrayal of the Cr(OH)_3_ adsorption mechanism, predominantly driven by vdW interactions, complemented by hydrogen bonds at critical stages, with steric effects playing a supplementary role. This granular insight is invaluable for the development of advanced adsorbent materials, enhancing the efficiency of wastewater treatment solutions.

#### 2.12.4. Topology Analysis

Bader’s QTAIM theory is widely utilized to probe the nature of interactions within a variety of molecular systems. It offers a detailed analysis of bonding through real-space functions, particularly electron density at bond critical points (BCPs) [64,65]. QTAIM’s ability to discern between strong and weak molecular interactions renders it indispensable for experimental researchers exploring host–guest molecular systems. In this study, the QTAIM analysis offers an insightful examination of the interactions between Cr(OH)_3_ and the kaolinite surface.

**Figure 19:**
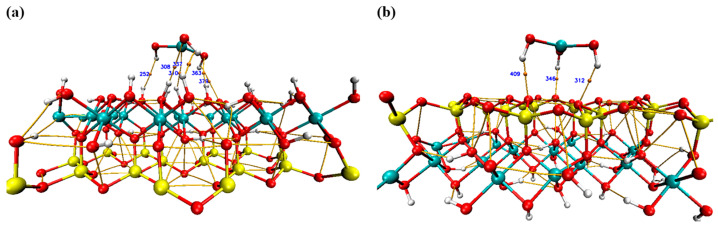
The QTAIM maps of the kaolinite surface attached to Cr(OH)_3_ in Si–O (**a**) and Al–O(H) (**b**) layers.

As illustrated in Table 9 and Figure 19, QTAIM reveals the complex bonding within the Cr(OH)_3_-kaolinite adsorption system, particularly within the Al–O(H) and Si–O layers. The analysis not only underscores the diversity in bond strength, as indicated by the electron density and Laplacian values at bond critical points (BCPs), but also delineates the character of these interactions, such as hydrogen bonding and van der Waals forces. For example, BCP 252 in the Al–O(H) layer reveals a significant interaction between Cr(OH)_3_ and kaolinite, characterized by its electron density value, indicative of a potential hydrogen bond crucial for adsorption [16]. This nuanced understanding of molecular interactions obtained from QTAIM metrics provides a robust framework for designing optimized kaolinite-based adsorbents for environmental applications, particularly for the removal of chromium pollutants from water. This research contributes novel perspectives to the field of adsorption science, paralleling the significance of kaolinite studies involving a variety of other compounds, such as amino alcohol [57], 2-phosphonobutane-1,2,4-tricarboxylic acid [66], ciprofloxacin [60], asphaltene [67], bisphenol A, and bisphenol S [59].

## 3. Materials and Methods

### 3.1. Kaolin Preparation

Tamazert kaolin, from the Tamazert region, situated 14 km from El-Milia in the Jijel region (Northeast Algeria), was provided by the Algerian Kaolin Society’s El-Milia kaolin complex (SOALKA, El-Milia, Algeria). KT2 is the commercial name for this compound. To raise the amount of kaolinite in this product, wet particle size separation was used. Kaolin was also exposed to the following physical treatments before to be used as an adsorbent:

- Drying with a Memmert oven (Schwabach, Germany) set to 110 °C;
- Grinding with a Rescht PM 100 electric grinder (San Diego, CA, USA);
- Sieving with sieves of precise diameter (0.06 mm);
- Storage in a desiccator.

### 3.2. Characterization of Kaolin

The XRD analysis was performed on a powder diffractometer with the Bragg–Brentano geometry (θ, 2θ configuration): BRUKER-AXE D8-ADVANCE model (Bruker, Billerica, MA, USA). A copper anticathode powered at 40 kV and 40 mA generated the X-rays. A curved graphite back monochromator separates the Cu radiation K_α1_ (K_α1_ = 1.5406 Å) and K_α2_ (K_α2_ = 1.54439 Å). The data were obtained over a 2θ range from 5 to 60° with a step of 0.04.

A PERKIN-ELMER spectrometer (Waltham, MA, USA) was used for the FTIR investigation of kaolin. The spectra were taken in the mid-infrared spectrum, with wave numbers (υ = 1/λ) ranging from 4000 to 400 cm^−1^ on pellets made by dispersing 4 mg of the support in 100 mg of KBr. HITACHI 4100S scanning electronic microscopy (SEM, Tokyo, Japan) with an analyzer EDS was used for the morphology study and the elementary analysis of the kaolin. The sample was ground as fine particles and mechanically dispersed on an electrically conductive carbon tape, which was placed on an aluminum disc. Kaolin particle size analysis was performed using a Malvern Mastersizer X Ver 2.15 laser particle sizer (Malvern Instruments Ltd., Malvern, UK). The measurements were carried out at a source wavelength of 2.40 mm and focal lengths of 300 and 45 mm, allowing for the analysis of particles with diameters ranging from 1 to 600 µm and 0.1 to 80 µm. Before measurement, the particles were dispersed in bidistilled water under the action of ultrasound for 30 s. A LEYBOLD HERAEUS LHS10 spectrometer (Cologne, Germany) was used for the X-ray photoelectron spectroscopy (XPS) analysis. The experiment was done in an ultravacuum (10^−9^ torr) to improve the resolution of the spectra and the sensitivity of the analysis and to avoid surface contamination. The aluminum K_α_ line (13 kV, 20 mA) was employed as an X-ray source, and electron energies were determined in the constant analyzer energy mode. The C 1s photoelectron line at 285.0 eV was used as the binding energy reference. The specific surface area was calculated using a N_2_ adsorption–desorption isotherm at 77.3 K. The sample was first degassed at 110 °C for 3 h at a low pressure using a Quanta chrome Nova Win2 instrument (Boynton Beach, FL, USA). The thermal analysis of kaolin was carried out using a TGA CETARAM 92-16 instrument (Lyon, France). The curves were measured in air between room temperature and 800 °C. The chosen temperature rise rate was 10 °C·min^−1^. The point of zero charge pH_PZC_ of the kaolin was established by a method described elsewhere [68].

This method consists of introducing a fixed mass of the support into 50 mL of a solution of potassium nitrate KNO_3_ (0.1 M), whose pH had been adjusted beforehand. The mixture was subjected to agitation (500 rpm) for 24 h in a cell thermostated at 22 °C. After filtration of the solution, the final pH was measured. The value of pH_PZC_ corresponds to the point pH_final_ = pH_initial_ obtained from the curve (pH_final_ − pH_initia_l) vs. pH_initial_.

To evaluate the zeta potential, suspensions were prepared by introducing a fixed mass of kaolin in the KCl solution (0.01 M), and the pH of the solutions was adjusted to the desired level using NaOH and HCl. Solutions were then subjected to agitation for 24 h at ambient temperature. After centrifugation (1200 rpm), the supernatant was recovered for measurement of the zeta potential by Zetamaster Malvern Instruments.

### 3.3. Reagents and Solutions

All the products used were of recognized analytical quality. Chemicals used were CrCl_3_·6H_2_O (98%, Sigma Aldrich, St. Louis, MO, USA), HNO_3_ (65%, Riedel-de Haen, Buchs, Switzerland), NaOH (99%, Fluka, Honeywell, Charlotte, NC, USA), KCl (99%, Fluka), and NaNO_3_ (99% EPR). A stock synthetic wastewater solution was prepared by dissolving 1000 mg of CrCl_3_·6H_2_O in 1 L of double distilled water. Different solutions of concentrations varying from 5 to 75 mg/L were obtained by dilution. HNO_3_ (0.1 M) and NaOH (0.1 M) solutions were used to adjust the solution’s pH, measured with a Hanna pH.211 pH meter.

### 3.4. Batch Adsorption Experiment

Both equilibrium and kinetic studies were carried out by mixing in beakers 1 g of kaolin with 100 mL of Cr(III) solution at various concentrations (5, 10, 20, 40, 50, and 75 mg/L) and fixed pH (pH = 5). The samples were put in a thermostat bath and stirred at a speed of 300 rpm. The solid–liquid separation was done using a Millipore filter (Billerica, MA, USA, 0.45 µm), and the aliquot concentrations were then evaluated. The pH effect was evaluated using a solution of 10 mg/L at 22 °C with a solid–liquid ratio fixed at 10 g/L and varying the pH from 1 to 11. Temperature effect was studied at 5, 10, 22, 30, and 40 °C and pH 5. The influence of the mass of the adsorbent was studied by mixing separately 0.1, 0.3, 0.5, 0.8, 1.2, and 1.4 g in 100 mL of a Cr(III) solution (10 mg/L) at 22 °C and pH 5. The quantification of Cr(III) in the samples was achieved by using a SHIMADZU 1601 UV–visible spectrophotometer (Kyoto, Japan) following a detailed protocol. Initially, Cr(III) ions in the solution were oxidized to Cr(VI) using a solution of potassium permanganate. The resultant Cr(VI) ions were then complexed with 1,5-diphenylcarbazide (DPC) in an acidic medium to form a colored complex, which is a widely recognized method for the colorimetric determination of Cr(VI), as outlined in the ASTM D1687-17 standard method [69]. The absorbance of this complex was measured, allowing for the accurate determination of the Cr(VI) concentration, which was subsequently used to calculate the equilibrium adsorption capacity *q* (mg/g) of the kaolin for Cr(III) (Equation (10)). The specific procedure for preparing the DPC complex, including the concentrations of DPC and the conditions under which the colorimetric reaction was conducted, were strictly followed as per the standard guidelines to ensure reproducibility and accuracy of the results.

(10) $$q=\frac{C_{0}-C_{e}}{m}\cdot V$$

where *C*_0_ and *C_e_* are the initial concentration and the concentration at equilibrium (mg/L), respectively; *V* is the volume of the solution (L); and *m* is the mass of the adsorbent (g).

Adsorption isotherms were modelled by classical adsorption models: two-parameter Langmuir model, Freundlich, Temkin, Elovich, and Dubinin–Radushkevich.

The classical Langmuir and Freundlich model equations are given by the Equations (11) and (12):

(11) $$q_{e}=\frac{q_{max}bC_{e}}{1+bC_{e}}$$

(12) $$q_{e}=K_{f}C_{e}^{1/n}$$

where *q_max_* (in mg/g) is the maximum adsorption capacity, *b* (in L/g) is the Langmuir equilibrium constant, and *K_f_* (in (mg/g)/(mg/L)^1/*n*^) and 1/*n* are the Freundlich constants.

The Elovich isotherm assumes that the adsorption takes place in multiple layers and is expressed by Equation (13):

(13) $$\frac{q_{e}}{q_{m}}=K_{E}C_{e}exp\frac{1}{n}$$

where *K_E_* is the Elovich constant and *q_m_* is the maximum capacity of Elovich.

The Temkin model is applicable in the case of heterogeneous surfaces and is represented as:

(14) $$q_{e}=B_{t}\ln\left(K_{t}C_{e}\right)$$

where *K_t_* (L/g) is the Temkin constant and *B_t_* is a constant related the heat of adsorption (kJ·mol^−1^).

The Dubinin–Radushkevich isotherm, which is based on the following expression, is frequently used to determine typical porosity:

(15) $$q_{e}=q_{m}\ln\left(K\varepsilon^{2}\right)$$

where *q_e_* is the equilibrium adsorption capacity (mg·g^−1^), *q_m_* is the adsorption capacity at saturation (mg·g^−1^), the constant *K* (mole^2^/kJ^2^) gives the mean free energy, and *ε* is the Polanyi potential given by the following expression:

(16) $$\varepsilon =RT\ln\left(1+\frac{1}{C_{e}}\right)$$

where *R* is the gas constant (8.314 J/mol·K) and *T* is the absolute temperature.

The pseudo-second order kinetic model is generally given by the following equation:

(17) $$\frac{{dq}_{t}}{dt}=k_{2}{\left(q_{e}-q_{t}\right)}^{2}$$

After integration, the linear form is given by Equation (18):

(18) $$\frac{t}{q_{t}}=\frac{1}{q_{e}^{2}k_{2}}+\frac{1}{q_{t}}t$$

where $q_{e}$ and $q_{t}$ are the equilibrium adsorption capacity and instantaneous adsorption capacity (mg/g), respectively, and $k_{2}$ is the kinetic rate.

### 3.5. DFT Study

The DFT study in this research employs the M06-2X [70] functional and the TZVP basis set [71], executed through the Turbomole software version 4.4.1 [72,73], to optimize and analyze the adsorption system. This study extends beyond conventional DFT analyses by incorporating advanced techniques like reduced density gradient (RDG) and the quantum theory of atoms in molecules (QTAIM) for a detailed investigation of non-covalent interactions (NCIs) within the system [74,75].

Additionally, the COSMO-RS method and orbital analysis were utilized to assess the reactivity and electronic properties of the system, focusing on the energies of the highest occupied molecular orbital (HOMO) and the lowest unoccupied molecular orbital (LUMO). The RDG analysis, crucial for identifying van der Waals interactions and other weak non-covalent forces [61,76,77], was complemented by NCI analyses performed using Multiwfn 3.8 packages [58]. These analyses were visualized through the visual molecular dynamics (VMD) [78] interface and Gnuplot software version 5.4 [79], offering a comprehensive perspective on the interaction dynamics within the adsorption system.

## 4. Conclusions

In this study, the efficacy of Algerian Tamazert kaolin as an adsorbent for Cr(III) removal from wastewater was rigorously evaluated. Characterization techniques, including XRD and FT-IR, confirmed the crystalline structure and kaolinite phase dominance, while EDS, XPS, and particle size analyses detailed the kaolin’s composition and micrometric particle presence. BET surface area measurements and the thermo-gravimetric analysis provided insights into the mesoporous nature and thermal stability of the clay, with zeta potential measurements indicating surface charge characteristics and particle stability in the solution.

The adsorption experiments demonstrated Tamazert kaolin’s high Cr(III) adsorption capacity, achieving 99.11% removal efficiency under optimal conditions. Influential parameters such as pH, initial concentration, adsorbent mass, and ionic strength were systematically explored, revealing the adsorption’s dependency on these factors. The process was found to be spontaneous and can be driven by a combination of physisorption and chemisorption, as evidenced by the thermodynamic study. Langmuir isotherm conformity suggested monolayer adsorption.

Complementing these findings, DFT computations and quantum chemical analyses, including COSMO-RS, molecular orbitals, IGM, RDG, and QTAIM, provided a molecular-level understanding of the adsorptive interactions. These theoretical tools, in conjunction with experimental data, have propelled biosorbent research forward, improving the understanding of adsorption mechanisms. This study underscores the significance of continuous innovation in adsorbent material development and regeneration techniques, contributing to sustainable industrial and environmental remediation efforts.

## Figures and Tables

**Table 1:** Intense diffraction peaks of the three phases.

Minerals	Kaolinite				Quartz	Muscovite		
Plan	0 0 1	0 0 2	2 0 1	131	1 0 1	0 0 2	0 0 4	1 1 4
d_hkl_ (Å)	7.15	3.56	2.56	2.34	3.34	10.03	4.98	3.23
2θ (°)	12.36	24.92	34.96	38.36	26.60	8.8	17.76	27.52

**Table 2:** Chemical composition of kaolin [34].

Compound	SiO_2_	Al_2_O_3_	Fe_2_O_3_	TiO_2_	CaO	MgO	K_2_O	Na_2_O	Ignition Loss
% weight	49.30	33.5	1.59	0.24	0.08	0.40	2.75	0.09	10.5

**Table 3:** Particle size distribution results.

Estimated Diameters (µm)	Focal Distance = 300 mm	Focal Distance = 45 mm
D_10_	2	1.59
D_50_	8.32	8.25
D_90_	32.01	33.68
D (3.2)	13.26	3.96

**Table 4:** Chemical composition of the surface of kaolin.

Element	Binding Energy (eV)	Atomic %
O 1s	532.3	71.88
Si 2p	103	8.31
Al 2p	74.8	3.59
C 1s	285	8.21
Fe 2p	712.4	3.24
K 2p	294.7	4.77

**Table 5:** Textural characteristics determined from N_2_ adsorption–desorption.

Method	Specific Surface Area (m^2^/g)	Total Pore Volume (cm^3^/g)	Average Pore Diameter (nm)
BET	18.376	0.09376	20.4

**Table 6:** Thermodynamic parameters relating to Cr(III) adsorption on kaolin.

T (K)	ΔG^0^ (kJ·mol^−1^)	ΔH^0^ (kJ·mol^−1^)	ΔS^0^ (J·K^−1^·mol^−1^)
295	−11.567	71.783	283.916
303	−14.244		
313	−16.606		

**Table 7:** Adsorption isotherm parameters.

**Models**	**Parameters**		***R* ^2^**
**Langmuir**	*q_m_* (mg/g)	*b* (mL/μg)	
Type I	6.571	1.977	0.9958
Type II	8.422	1.151	0.9807
**Freundlich**	*K_f_* (mg/g) (μg/mL)	1/*n*	0.9835
	3.603	0.56	
**Temkin**	*K_Tem_* (L/g)	*B_T_* (kJ/mol)	0.9495
	21.502	1.624	
**Elovich**	*q_m_* (mg/g)	*K_E_* (L/mg)	0.9485
	3.618	2.031	
**Dubinin–Radushkevich**	*q_m_* (mg/g)	*K* (mol^2^/kJ^2^)	0.9262
	5.601	0.041	

**Table 8:** Comparative analysis of maximum Langmuir adsorption capacities of Cr(III) for various adsorbents.

Adsorbent	Initial pH	Langmuir Adsorption Capacity q_max_ (mg/g)	Ref.
Pomelo fruit peel	5	11.3–12.4	Van-Phuc Dinh et al. [50]
Aluminum oxide hydroxide	3.8	3.36	Bedemo et al. [51]
Palm flower (2 types)	4.5	6.24	Elangovan et al. [52]
	4.5	1.41	
Kaolinite Clay	4.5–5.5	2–3.44	Turan et al. [45]
Activated carbon (2 types)	2–6	12.2	Mohan et al. [39]
	2–6	39.56	
Activated carbon (4 types)	-	7.08	Rivera-Utrilla et al. [41]
	-	3.52	
	-	13.31	
	-	10.52	
Expanded perlite	4.5	0.73	Chakir et al. [53]
Kaolinite clay	5	8.422	This study

**Table 9:** Topological features of interaction sites (in atomic units) at selected bond critical points (BCPs).

BCP	X–Y	ρ(r)	∇^2^ρ(r)	G(r)	V(r)	E_HB_	|V(r)|/G(r)	H(r)
**Cr(OH)_3_^…^Al–O(H) layer**								
252	32(H)–142(H)	0.003220	0.010300	0.001910	−0.001260	−0.000629	−0.659686	0.000543
308	64(H)–140(O)	0.007270	0.016300	0.003490	−0.002920	−0.001460	−0.836676	0.000593
310	33(H)–137(Cr)	0.005640	0.007260	0.001720	−0.001630	−0.000816	−0.947674	−0.000164
337	67(H)–143(H)	0.008760	0.026200	0.005440	−0.004320	−0.002160	−0.794118	0.001245
363	138(O)–130(H)	0.006970	0.016300	0.003450	−0.002820	−0.001410	−0.817391	0.000688
379	141(H)–94(O)	0.003480	0.012300	0.002280	−0.001480	−0.000742	−0.649123	0.000752
**Cr(OH)_3_^…^Si–O layer**								
312	142(H)–81(O)	0.011400	0.033100	0.007160	−0.006060	−0.003030	−0.846369	0.001401
346	50(O)–143(H)	0.013900	0.042100	0.009330	−0.008120	−0.004060	−0.870311	0.001570
409	114(O)–141(H)	0.013300	0.039800	0.008780	−0.007610	−0.003800	−0.866743	0.001648

## Data Availability

Data available upon request.
